# A second solvatomorph of poly[[μ_4_-*N*,*N*′-(1,3,5-oxadiazinane-3,5-di­yl)bis­(carbamoyl­methano­ato)]nickel(II)dipotassium]: crystal structure, Hirshfeld surface analysis and semi-empirical geometry optimization

**DOI:** 10.1107/S2056989021011774

**Published:** 2021-11-12

**Authors:** Maksym O. Plutenko, Matti Haukka, Alina O. Husak, Irina A. Golenya, Nurullo U. Mulloev

**Affiliations:** a Department of Chemistry, National Taras Shevchenko University, Volodymyrska, Street 64, 01601 Kyiv, Ukraine; b Department of Chemistry, University of Jyvaskyla, P.O. Box 35, FI-40014 Jyvaskyla, Finland; c PBMR Labs Ukraine, Murmanska 1, 02094 Kiev, Ukraine; d The Faculty of Physics, Tajik National University, Rudaki Avenue 17, 734025 Dushanbe, Tajikistan

**Keywords:** nickel(II) complex, template reaction, pseudomacrocyclic ligand, hydrazide-based ligand, SHAPE analysis, Hirshfeld surface analysis, semi-empirical geometry optimization, crystal structure

## Abstract

The title compound, poly[tri­aqua­bis­[μ_4_-*N*,*N*′-(1,3,5-oxadiazinane-3,5-di­yl)bis(carbamoyl­methano­ato)]dinickel(II)tetra­potassium], [K_4_Ni_2_(C_7_H_6_N_4_O_7_)_2_(H_2_O)_3_]_*n*_, is a second solvatomorph of poly[(μ_4_-*N*,*N*′-(1,3,5-oxadiazinane-3,5-di­yl)bis­(carbamoyl­methano­ato)nickel(II)dipotassium] reported previously [Plutenko *et al.* (2021 ▸). *Acta Cryst.* E**77**, 298–304]. The asymmetric unit of the title compound includes two structurally independent complex anions [Ni(C_7_H_6_N_4_O_7_)]^2−^, which exhibit an L-shaped geometry and consist of two almost flat fragments perpendicular to one another: the 1,3,5-oxadiazinane fragment and the fragment including other atoms of the anion. The central Ni atom is in a square-planar N_2_O_2_ coordination arrangement formed by two amide N and two carboxyl­ate O atoms. In the crystal, the title compound forms a layered structure in which layers of negatively charged complex anions and positively charged potassium cations are stacked along the *a*-axis direction. The polymeric framework is stabilized by a system of hydrogen-bonding inter­actions in which the water mol­ecules act as donors and the carb­oxy­lic, amide and water O atoms act as acceptors.

## Abstract 2

The title compound is a second solvatomorph of the earlier reported compound. The complex nickel(II) anions exhibit an L-shaped geometry. The central Ni atom is in a square-planar N_2_O_2_ coordination arrangement. In crystal, the complex nickel(II) anions and the potassium cations form layers stacked along the *a*-axis direction.

## Chemical context

In 1976, the products of the metal-templated reaction of hydrazide and aldehyde were separated and structurally described (Clark *et al.*, 1976 ▸). It was further shown that such a synthetic strategy makes it possible to obtain complexes with 3*d* metals in high oxidation states. In particular, there are several works devoted to copper(III) complexes obtained by this method (Oliver & Waters, 1982 ▸; Fritsky *et al.*, 1998 ▸, 2006 ▸). Moreover, the preparation of an unprecedentedly stable iron(IV) clathrochelate complex was reported (Tomyn *et al.*, 2017 ▸). Some such compounds are promising redox catalysts, as has been shown by Pap *et al.* (2011 ▸) and Shylin *et al.* (2019 ▸). Thus, the study of the conditions and peculiarities of hydrazide-aldehyde template inter­actions, as well as the isolation and characterization of their products, is an important task in modern coordination chemistry.

**Figure 1:**
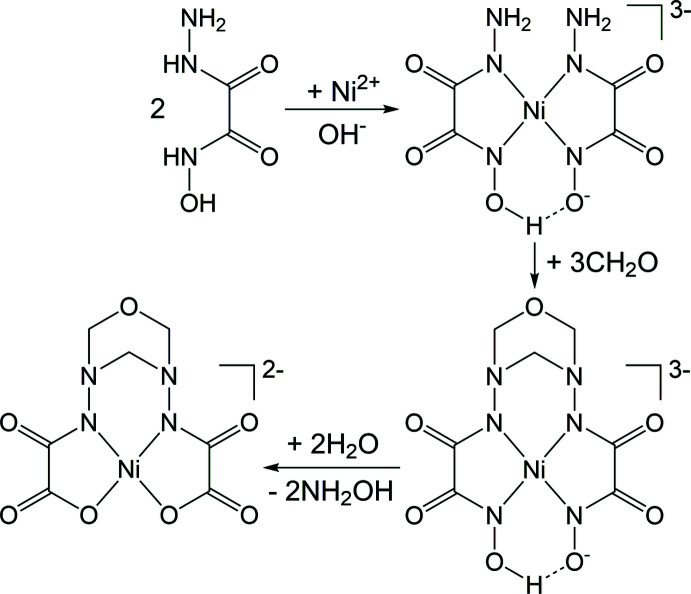
A plausible mechanism for the formation of the [Ni(*L*-2H)]^2–^ complex anion.

This work is a continuation of our investigation of the inter­action of oxalohydrazide­hydroxamic acid with formaldehyde and nickel(II) salts. Here we report the crystal structure of the title compound poly[tri­aqua­bis­[μ_4_-*N*,*N*′-(1,3,5-oxadiazinane-3,5-di­yl)bis­(carbamoyl­methano­ato)]dinick­el(II)tetra­potassium] [(2K_2_[Ni(*L*-2H)]·3H_2_O)_*n*_, **2**], which is the solvatomorph of the earlier published (Plutenko *et al.*, 2021 ▸) complex poly[penta­aqua­bis­[μ_*n*_-*N*,*N*′-(1,3,5-oxadiaz­inane-3,5-di­yl)bis­(carbamoyl­methano­ato)]nickel(II)tetra­pot­assium], [(2K_2_[Ni(*L*-2H)]·4.8H_2_O)_*n*_, **1**, H_2_ *L* = *N*,*N*′-(1,3,5-oxadiazinane-3,5-di­yl)bis­(amino­oxo­acetic acid)]. Both compounds can be obtained in a similar fashion as the result of a one-pot template reaction (see Fig. 1 ▸). 
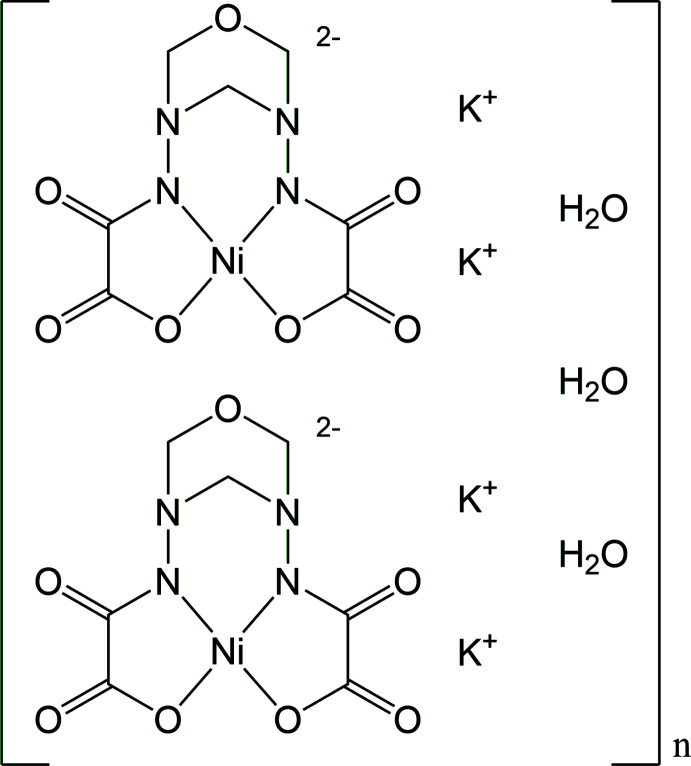


## Structural commentary

**Figure 2:**
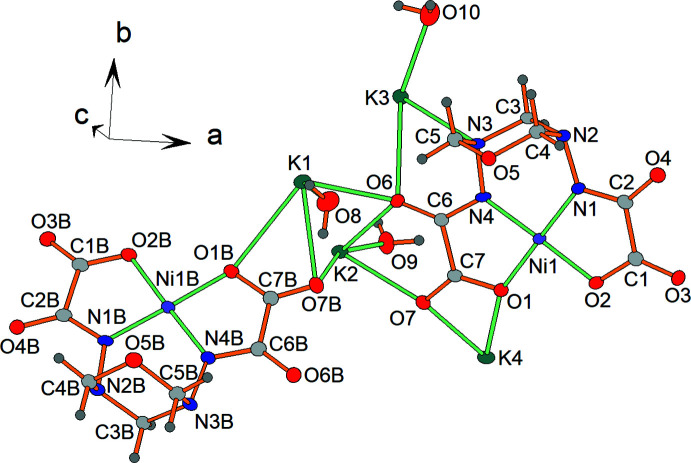
The asymmetric unit of **2** with displacement ellipsoids shown at the 50% probability level.

The title compound, **2**, (2K_2_[Ni(*L*-2H)]·3H_2_O)_*n*_, crystallizes in space group *P*2_1_/*c*, while the previously reported compound **1**, (2K_2_[Ni(*L*-2H)]·4.8H_2_O)_*n*_, crystallizes in *Pbca*. Similarly to **1**, the asymmetric unit of **2** (Fig. 2 ▸) includes two structurally independent complex anions [Ni(*L*-2H)]^2–^ (namely *A* and *B*, which contain Ni1 and Ni1*B*, respectively). In addition, the unit cell of **2** also contains four potassium cations and three solvent water mol­ecules.

Similarly to **1**, the complex anion [Ni(*L*-2H)]^2−^ has an L-shaped geometry and consists of two almost flat fragments perpendicular to one another: the 1,3,5-oxadiazinane fragment and the fragment including other atoms of the anion. The dihedral angles between the mean planes formed by the non-hydrogen atoms of these fragments are 95.06 (8) and 94.06 (8)° for Ni1 and Ni1*B*, respectively. The ligand mol­ecule is coordinated in a tetra­dentate {O_carbox­yl_,N_amide_,N_amide_,O_carbox­yl_}-mode. The central atom of the complex anion exhib­its a square-planar coordination arrangement with the N_2_O_2_ chromophore. The deviation of the Ni^II^ atom from the mean plane defined by the donor atoms is 0.0073 (13) and 0.0330 (12) Å for Ni1 and Ni1*B*, respectively.

The Ni—N bond distances are in the range 1.836 (3)–1.849 (3) Å and Ni–O bond lengths are 1.877 (2)–1.897 (2) Å, which is typical for square-planar nickel complexes with similar ligands (Fritsky *et al.*, 1998 ▸) and close to the Ni—N and Ni–O bond distances of **1**. The O—*M*—O′, O—*M*—N and N—*M*—N′ bond angles have typical values for a square-planar arrangement. The bite angles O1—Ni1—N4, N1—Ni1—O2 and N1—Ni1—N4 deviate from 90°, which is the result of the formation of the five-membered chelate rings. The N—N′, N—C and C—O bond lengths of the ligand have typical values for coordinated deprotonated hydrazide and carboxyl groups.

## Supra­molecular features

**Figure 3:**
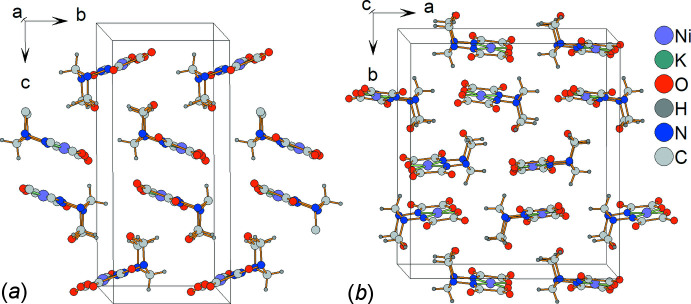
Layers formed by the nickel(II) complex anions [Ni(*L*-2H)]^2–^ in the crystals of (*a*) compound **2** and (*b*) compound **1**.

**Figure 4:**
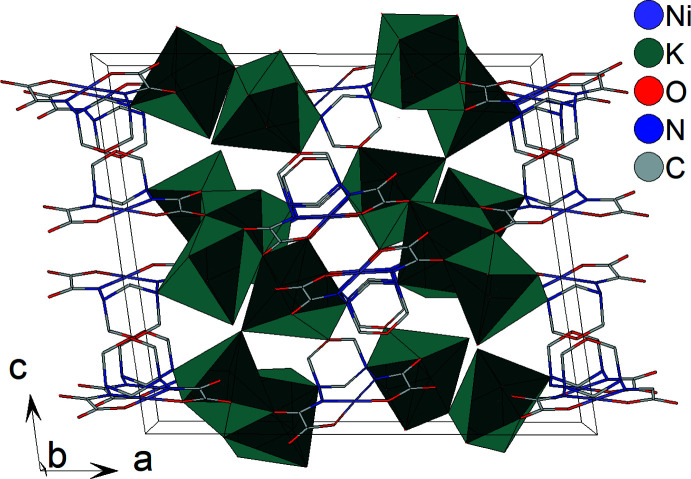
Crystal packing of the title compound in a stick model, showing the coordination polyhedra of the potassium cations. H atoms are omitted for clarity.

In the crystal, the nickel(II) complex anions [Ni(*L*-2H)]^2−^ form layers parallel to the *bc* plane (Fig. 3 ▸ *a*). Neighbouring complex anion layers are sandwiched by layers of potassium counter-cations (Fig. 4 ▸). Thus, negatively charged complex anion layers and positively charged potassium cationic layers are stacked along the *a*-axis direction. It is useful to note that a similar layered structure motif was observed in the crystal of the previously published compound **1**. However, in the crystal of **1** the NiN_2_O_2_ plane is almost perpendicular to the complex anion layer plane (Fig. 3 ▸ *b*): the angle between NiN_2_O_2_ and the *ab* plane is 84.43 (4) and 85.03 (5)° for Ni1 and Ni1*B*, respectively. In contrast, in the crystal of **2** the angle between NiN_2_O_2_ and the *bc* plane is 78.30 (8) and 86.29 (7)° for Ni1 and Ni1*B*, respectively.

The demarcation of bonded and non-bonded K—*X* inter­actions (*X* = N or O) is still an unclear and debatable problem (Alvarez, 2013 ▸). Therefore, the criteria of such demarcation used in this paper need to be detailed. Based on the aforementioned publication (Alvarez, 2013 ▸), we propose 3.7 Å as the maximal distance for K—N bonds. Recently, it was shown (Gagné & Hawthorne, 2016 ▸) that K—O main and maximal bond distances depend on the coordination number of K. The results of this work permits 3.4, 3.5 and 3.6 Å to be proposed as the maximal distances for K—O bonds in the case of potassium coordination numbers 7, 8 and 9, respectively. In addition, K⋯N_amide_ inter­actions were determined as non-bonding because the existence of such bonds would lead to the presence of unstable three-membered KN_amide_N_oxadiazinane_ rings with extremely small N—K—N′ angles.

The potassium cations are bound to the nickel(II) complex anions through the carb­oxy­lic O atoms (K4) the carb­oxy­lic and the amide O atoms (K1, K2) or through the amide O and the oxadiazinane N atoms (K3). In addition, the potassium cations have contacts with the O atoms of water mol­ecules, with the amide and the carb­oxy­lic O atoms, and with the oxadiazinane O and N atoms of neighbouring complex anions. The K1 and K2 cations exhibit an O_6_N coordination, while the K3 and K4 cations exhibit O_8_N and O_7_N coordinations, respectively.

**Figure 5:**
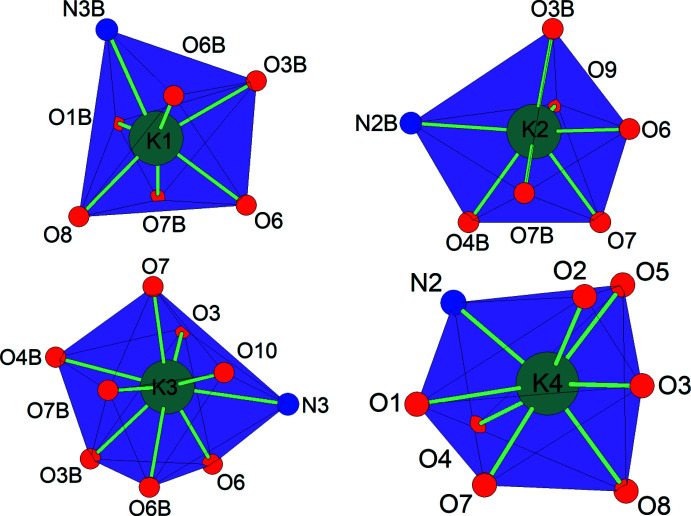
Polyhedral views of the coordination environments for the potassium cations.

For an evaluation of the coordination geometry of each potassium cation, *SHAPE 2.1* software (Llunell *et al.*, 2013 ▸) was used. A SHAPE analysis of the potassium coordination sphere (Table 1 ▸, Fig. 5 ▸) yields the lowest continuous shape measure (CShM) value for a distorted penta­gonal bipyramid (5.142 for K1 and 3.122 for K2), a distorted muffin (3.691 for K3) and a distorted triangular dodeca­hedron (5.187 for K4). For K4, comparable CShM values were obtained for a square anti­prism (5.463).

The polyhedra around the neighbouring potassium cations are connected with each other through common vertices (K1 with K3, K1 with K4, K2 with K4), edges (K3 with K4) and faces (K1 with K2, K1 with K3, K2 with K3). The K—O bond lengths are in the range 2.628 (2)–3.271 (3) Å, K—N 2.887 (3)–3.025 (3) Å, which is close to those reported for the structures of related carboxyl­ate and amide complexes (Fritsky *et al.*, 1998 ▸; Mokhir *et al.*, 2002 ▸).

**Figure 6:**
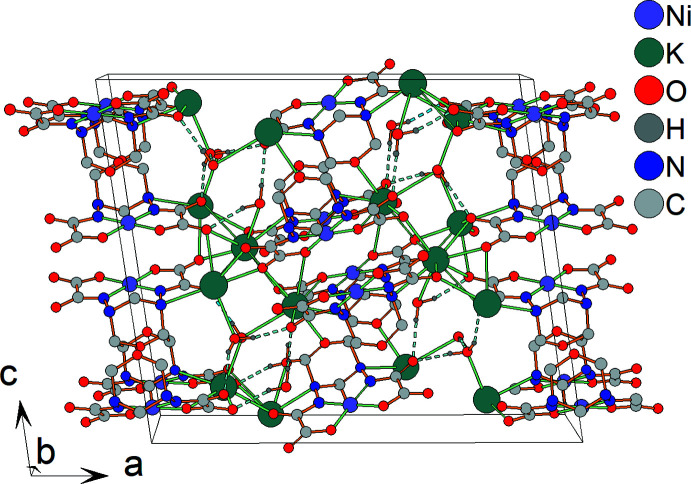
Crystal packing of the title compound. C—H hydrogen atoms are omitted for clarity. Hydrogen bonds are indicated by dashed lines.

The polymeric framework of **2** is stabilized by an extensive system of hydrogen-bonding inter­actions in which the water mol­ecules act as donors and the carb­oxy­lic, the amide and the water O atoms act as acceptors (Table 2 ▸). Similarly to **1**, the hydrogen bonds are localized mainly at the potassium cation layers (Fig. 6 ▸). Moreover, in comparison to **1**, the unit cell of **2** contains a smaller number of water mol­ecules, which causes a smaller number of hydrogen-bond inter­actions in the crystal structure.

## Hirshfeld analysis

The Hirshfeld surface analysis (Spackman & Jayatilaka, 2009 ▸) and the associated two-dimensional fingerprint plots (McKinnon *et al.*, 2007 ▸) were performed with *CrystalExplorer17* (Turner *et al.*, 2017 ▸). The Hirshfeld surfaces of the complex anions are colour-mapped with the normalized contact distance (*d_norm_*) from red (distances shorter than the sum of the van der Waals radii) through white to blue (distances longer than the sum of the van der Waals radii).

**Figure 7:**
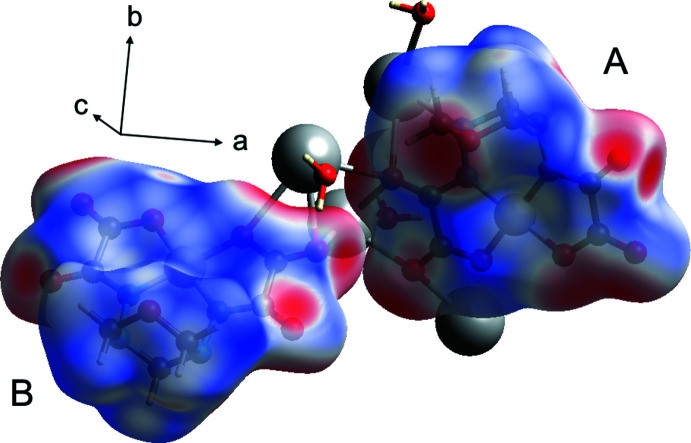
The Hirshfeld surfaces of the Ni1 (A) and Ni1*B* (B) complex anions mapped over *d*_norm_.

The Hirshfeld surface of the title compound is mapped over *d_norm_*, in the colour ranges −0.6388 to 0.9164 a.u. and −0.6768 to 0.7286 a.u. for Ni1 and Ni1*B* complex anions, respectively (Fig. 7 ▸). Similarly to **1**, the complex anions of **2** are connected to the other elements of the crystal packing mainly *via* the amide and the carb­oxy­lic O atoms. However, in contrast to **1**, one of the oxadiazinane O atoms of **2** is also involved in inter­molecular bond formation.

**Figure 8:**
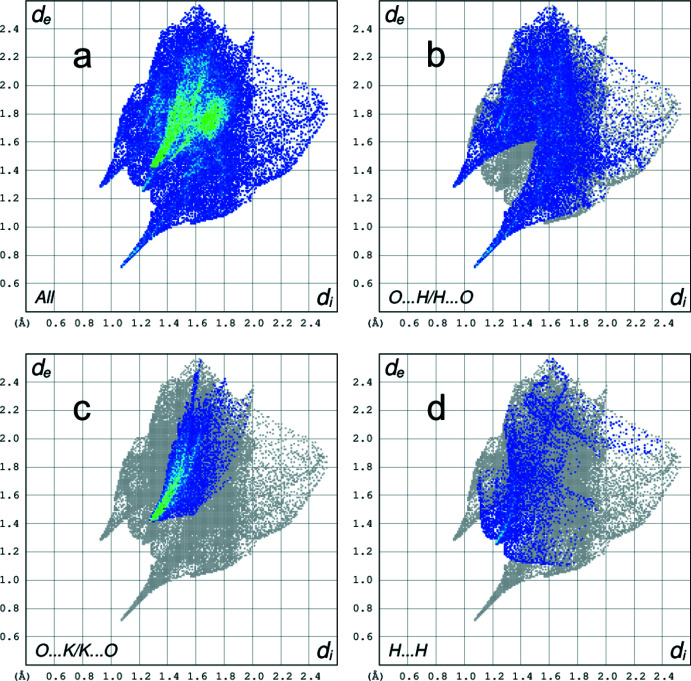
(*a*) Full two-dimensional fingerprint plot of the Ni1 complex anion and those delineated into (*b*) O⋯H/H⋯O (36.9%) (*c*) O⋯K/K⋯O (20.9%) and (*d*) H⋯H (10.4%) contacts.

A fingerprint plot delineated into specific inter­atomic contacts contains information related to specific inter­molecular inter­actions. The blue colour refers to the frequency of occurrence of the (*d*_i_, *d*_e_) pair with the full fingerprint plot outlined in gray. Fig. 8 ▸ *a* and 9 ▸ *a* show the two-dimensional fingerprint plots of the sum of the contacts contributing to the Hirshfeld surface represented in normal mode for the Ni1 and Ni1*B* complex anions, respectively.

The most significant contribution to the Hirshfeld surface is from O⋯H/H⋯O contacts (36.9% and 38.7% for the Ni1 and Ni1*B* complex anions, respectively; Fig. 8 ▸ *b* and 9 ▸ *b*). In addition, O⋯K/K⋯O (20.9% and 18.2% for the Ni1 and Ni1*B* complex anions; Fig. 8 ▸ *c* and 9 ▸ *c*) and H⋯H (10.4% and 13.1% for the Ni1 and Ni1*B* complex anions, respectively; Fig. 8 ▸ *d* and 9*d*) make very significant contributions to the total Hirshfeld surface. This indicates that there are more K⋯O contacts and fewer O⋯H contacts compared to the crystal of **1**.

## Geometry optimization

The searching of computationally ‘cheap’ but still sufficiently accurate methods of transition-metal complex geometry optimization is an important task of modern computational chemistry. The geometry optimization calculations were carried out with three semi-empirical methods: PM7, DFTB and GFN2-xTB. The PM7 (Stewart, 2013 ▸) calculations were performed with *MOPAC2016* software (Stewart, 2016 ▸). The DFTB calculations were carried out with the *DFTB+* software package (Hourahine *et al.*, 2020 ▸) using the ‘mio-1-1’ (Elstner *et al.*, 1998 ▸) and the ‘trans3d-0-1’ (Zheng *et al.*, 2007 ▸) Slater–Koster parameterization sets. The GFN2-xTB (Bannwarth *et al.*, 2019 ▸) calculations were applied with *xtb 6.4* package (Grimme, 2019 ▸). The geometry of the Ni1 complex anion obtained from the crystal structure was used as the starting geometry for the calculations.

**Figure 10:**
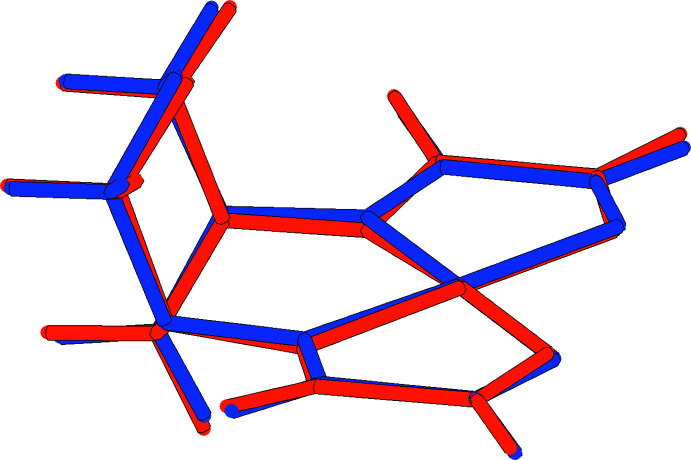
Structural overlay between the experimental (blue) and optimized (orange) structures.

In general, for all described semi-empirical methods, the calculated geometric parameters of the oxadiazinane ring are in reasonable agreement with experimental values (see Table 3 ▸). On the other hand, the accuracy of the non-oxadiazinane fragment geometry prediction varies greatly depending on the method. The worst agreement with experiments is from the PM7 method, mainly because of the pyramidalization of the amide nitro­gen atom (Table 3 ▸). Such non-planarity of the amide fragment is a well-known problem of the PMx methods (Feigel & Strassner, 1993 ▸). In contrast, the DFTB method predicts the amide geometric parameters with high accuracy but demonstrates longer than experimental carboxyl­ate C—O bonds and a slight tetra­gonal distortion of the nickel(II) coordination polyhedra (Table 3 ▸). The best results were obtained with the GFN2-xTB method for which the calculated geometric parameters correlate nicely with experimental values (Table 3 ▸). The maximal difference between the calculated and the experimental bond lengths concerns the C—O lengths (shorter than the experimental values within 0.024–0.033 Å). A superimposed analysis of the Ni1 complex anion with its optimized structure gives an RMSD of 0.131 Å (Fig. 10 ▸). Thus, the GFN2-xTB method is a promising geometry prediction method for transition-metal complexes based on hydrazide and carboxyl­ate ligands.

## Database survey

A search in the Cambridge Structural Database (CSD version 5.39, update of May 2018; Groom *et al.*, 2016 ▸) resulted in 11 hits dealing with 3*d*-metal complexes with macrocyclic or pseudo-macrocyclic ligands formed by template binding of several hydrazide groups by formaldehyde mol­ecules. These complexes contain the following 3*d* metals: Ni^II^ (Fritsky *et al.*, 1998 ▸), Cu^II^ (Clark *et al.*, 1976 ▸; Fritsky *et al.*, 2006 ▸), Cu^III^ (Oliver & Waters, 1982 ▸; Fritsky *et al.*, 1998 ▸, Fritsky *et al.*, 2006 ▸) and Fe^IV^ (Tomyn *et al.*, 2017 ▸). Thus, such macrocyclic and pseudo-macrocyclic ligand systems exhibit a tendency to stabilize the high oxidation states of 3*d* metals.

## Synthesis and crystallization

A solution of Ni(ClO_4_)_2_·6H_2_O (0.091 g, 0.25 mmol) in 5 ml of water was added to a warm solution of oxalohydrazide­hydroxamic acid (0.06 g, 0.5 mmol) in 5 ml of water. The resulting light-green mixture was stirred with heating (320–330 K) for 20 min and then 1 ml of 4*M* KOH solution was added. As a result, the colour of the solution changed to pink. After 5 min of stirring, 0.03 g of the paraformaldehyde (1 mmol) was added and stirring with heating (323–333 K) was continued for 30 min. The resulting orange solution was left for crystallization by slow evaporation in air. After one week, orange crystals of **2** suitable for X-ray diffraction studies were obtained. The crystals were filtered off, washed with diethyl ether and dried in the air. Yield 0.044 g (42%). Elemental analysis for C_14_H_18_N_8_O_17_K_4_Ni_2_ (mol. mass 844.12), calculated, %: C 19.92; H 2.15; N 13.27; Found, %: C 19.69; H 2.16; N 13.11. UV–vis (H_2_O), λ_max_ (ɛ, mol^−1^ dm^3^ cm^−1^): 520 nm (1380). IR (KBr, cm^−1^): 3420 *br* ν(O–H) stretch, 2981, 2910, 2860 ν(C—H) stretch, 1643 (*vs*) ν(C=O) amide I, 1590 ν_as_(COO^−^), 1435 ν_s_(COO^−^).

## Refinement

Crystal data, data collection and structure refinement details are summarized in Table 4 ▸. H atoms were positioned geom­etrically (O—H = 0.85–0.88, C—H = 0.99 Å) and refined as riding with *U*_iso_(H) = 1.2 *U*_eq_(O, C).

## Supplementary Material

Crystal structure: contains datablock(s) I. DOI: 10.1107/S2056989021011774/tx2044sup1.cif

Structure factors: contains datablock(s) I. DOI: 10.1107/S2056989021011774/tx2044Isup2.hkl

Supporting information file. DOI: 10.1107/S2056989021011774/tx2044Isup3.cdx

CCDC reference: 2120378

Additional supporting information:  crystallographic information; 3D view; checkCIF report

## Figures and Tables

**Figure 9:**
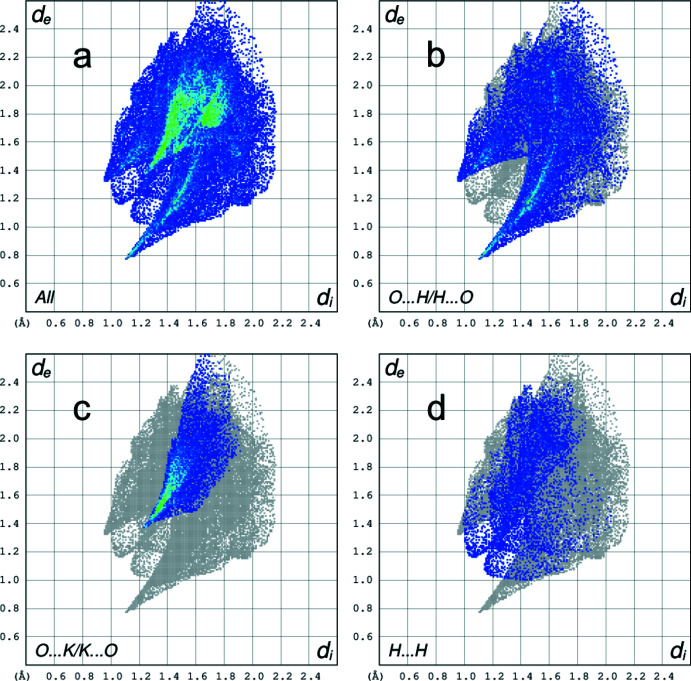
(*a*) Full two-dimensional fingerprint plot of the Ni1*B* complex anion and those delineated into (*b*) O⋯H/H⋯O (38.7%) (*c*) O⋯K/K⋯O (18.2%) and (*d*) H⋯H (13.1%) contacts.

**Table 1:** Values for continuous shapes measures (CShM) of the polyhedra centred by the potassium cations

Shape	CShM	
	K1	K2
Heptagon (*D*7*h*)	28.515	29.484
Hexagonal pyramid (*C*6*v*)	17.225	20.349
Penta­gonal bipyramid (*D*5*h*)	5.142	3.122
Capped octa­hedron (*C*3*v*)	7.539	7.840
Capped trigonal prism (*C*2*v*)	6.374	5.639
Johnson penta­gonal bipyramid J13 (*D*5*h*)	8.789	6.943
Johnson elongated triangular pyramid J7 (*C*3*v*)	16.352	20.453
	K3	
Enneagon (*D*9*h*)	32.593	
Octa­gonal pyramid (*C*8*v*)	23.087	
Heptagonal bipyramid (*D*7*h*)	14.962	
Johnson triangular cupola J3 (*C*3*v*)	12.759	
Capped cube J8 (*C*4*v*)	9.046	
Spherical-relaxed capped cube (*C*4*v*)	7.600	
Capped square anti­prism J10 (*C*4*v*)	6.360	
Spherical capped square anti­prism (*C*4*v*)	5.020	
Tricapped trigonal prism J51 (*D*3*h*)	6.694	
Spherical tricapped trigonal prism (*D*3*h*)	5.698	
Tridiminished icosa­hedron J63 (*C*3*v*)	11.379	
Hula-hoop (*C*2*v*)	6.577	
Muffin (*Cs*)	3.691	
	K4	
Octa­gon (*D*8*h*)	33.086	
Heptagonal pyramid (*C*7*v*)	18.988	
Hexagonal bipyramid (*D*6*h*)	14.426	
Cube (*Oh*)	10.884	
Square anti­prism (*D*4*d*)	5.463	
Triangular dodeca­hedron (*D*2*d*)	5.187	
Johnson gyrobifastigium J26 (*D*2*d*)	11.775	
Johnson elongated triangular bipyramid J14 (*D*3*h*)	26.080	
Biaugmented trigonal prism J50 (*C*2*v*)	6.413	
Biaugmented trigonal prism (*C*2*v*)	6.587	
Snub diphenoid J84 (*D*2*d*)	7.862	
Triakis tetra­hedron (*Td*)	11.175	
Elongated trigonal bipyramid (*D*3*h*)	20.295	

**Table 2:** Hydrogen-bond geometry (Å, °)

*D*—H⋯*A*	*D*—H	H⋯*A*	*D*⋯*A*	*D*—H⋯*A*
O8—H8*O*⋯O9^i^	0.85	2.02	2.869 (4)	173
O8—H8*P*⋯O4*B* ^ii^	0.85	2.01	2.858 (3)	166
O9—H9*P*⋯O4^iii^	0.86	1.91	2.722 (3)	157
O9—H9*O*⋯O6*B* ^iv^	0.86	2.07	2.864 (3)	153
O10—H10*P*⋯O4^v^	0.88	2.02	2.887 (3)	168
O10—H10*O*⋯O7*B* ^vi^	0.87	2.04	2.882 (3)	164

Symmetry codes: (i) x, -y+{\script{1\over 2}}, z+{\script{1\over 2}}; (ii) -x, y+{\script{1\over 2}}, -z+{\script{1\over 2}}; (iii) -x+1, -y+1, -z; (iv) x, -y+{\script{1\over 2}}, z-{\script{1\over 2}}; (v) -x+1, y+{\script{1\over 2}}, -z+{\script{1\over 2}}; (vi) x, y+1, z.

**Table 3:** Comparison of selected geometric data (A,°; mean values) for the Ni1 complex anion from calculated and X-ray data

Geometric parameter	X-ray	PM7	DFTB	GFN2-xTB
Oxadiazinane ring				
C—O	1.434	1.413	1.467	1.410
C—N	1.463	1.489	1.463	1.452
Carboxyl­ate moiety				
C—O	1.287	1.276	1.451	1.260
C=O	1.233	1.224	1.196	1.208
Hydrazide moiety				
C—O	1.249	1.232	1.227	1.216
C—N	1.321	1.357	1.393	1.332
N—N	1.432	1.413	1.413	1.415
C—N_amide_—Ni—N_oxadiazine_	175.74	133.89	169.00	162.81
Ni coordination arrangement				
Ni—O	1.892	1.776	1.780	1.871
Ni—N	1.840	1.955	1.974	1.871
O—Ni—N chelate	85.24	93.35	81.32	82.94
O—Ni—N non-chelate	178.29	173.19	162.52	176.77
N—Ni—N	85.53	88.09	90.73	94.40

**Table 4:** Experimental details

Crystal data	
Chemical formula	[K_4_Ni_2_(C_7_H_6_N_4_O_7_)_2_(H_2_O)_3_]
*M*_r_	844.18
Crystal system, space group	Monoclinic, *P*2_1_/*c*
Temperature (K)	100
*a*, *b*, *c* (Å)	20.3825 (5), 7.7039 (3), 17.3078 (6)
β (°)	98.240 (2)
*V* (Å^3^)	2689.69 (16)
*Z*	4
Radiation type	Mo *K*α
μ (mm^−1^)	2.12
Crystal size (mm)	0.15 × 0.09 × 0.08

Data collection	
Diffractometer	Bruker Kappa APEXII CCD
Absorption correction	Multi-scan (*SADABS*; Sheldrick, 2008 ▸)
*T*_min_, *T*_max_	0.746, 0.842
No. of measured, independent and observed [*I* > 2σ(*I*)] reflections	25068, 6148, 5118
*R*_int_	0.043
(sin θ/λ)_max_ (Å^−1^)	0.650

Refinement	
*R*[*F* ^2^ > 2σ(*F* ^2^)], *wR*(*F* ^2^), *S*	0.041, 0.082, 1.14
No. of reflections	6148
No. of parameters	406
H-atom treatment	H-atom parameters constrained
Δρ_max_, Δρ_min_ (e Å^−3^)	0.63, −0.45

Computer programs: *COLLECT* (Bruker, 2008 ▸), *DENZO*/*SCALEPACK* (Otwinowski & Minor, 1997 ▸), *SUPERFLIP* (Palatinus & Chapuis, 2007 ▸), *SHELXL2018/1* (Sheldrick, 2015 ▸), *DIAMOND* (Brandenburg, 2009 ▸) and *SHELXL97* (Sheldrick, 2008 ▸).

## supplementary crystallographic information

### Crystal data

**Table:**

[K_4_Ni_2_(C_7_H_6_N_4_O_7_)_2_(H_2_O)_3_]	*F*(000) = 1704
*M**_r_* = 844.18	*D*_x_ = 2.085 Mg m^−^^3^
Monoclinic, *P*2_1_/*c*	Mo *K*α radiation, λ = 0.71073 Å
*a* = 20.3825 (5) Å	Cell parameters from 12179 reflections
*b* = 7.7039 (3) Å	θ = 1.0–30.0°
*c* = 17.3078 (6) Å	µ = 2.12 mm^−^^1^
β = 98.240 (2)°	*T* = 100 K
*V* = 2689.69 (16) Å^3^	Orange, block
*Z* = 4	0.15 × 0.09 × 0.08 mm

### Data collection

**Table:**

Bruker Kappa APEXII CCD diffractometer	6148 independent reflections
Radiation source: fine-focus sealed tube	5118 reflections with *I* > 2σ(*I*)
Horizontally mounted graphite crystal monochromator	*R*_int_ = 0.043
Detector resolution: 16 pixels mm^-1^	θ_max_ = 27.5°, θ_min_ = 2.5°
φ scans and ω scans with κ offset	*h* = −26→26
Absorption correction: multi-scan (SADABS; Sheldrick, 2008)	*k* = −10→10
*T*_min_ = 0.746, *T*_max_ = 0.842	*l* = −22→22
25068 measured reflections	

### Refinement

**Table:**

Refinement on *F*^2^	0 restraints
Least-squares matrix: full	Hydrogen site location: mixed
*R*[*F*^2^ > 2σ(*F*^2^)] = 0.041	H-atom parameters constrained
*wR*(*F*^2^) = 0.082	*w* = 1/[σ^2^(*F*_o_^2^) + 8.0539*P*] where *P* = (*F*_o_^2^ + 2*F*_c_^2^)/3
*S* = 1.14	(Δ/σ)_max_ < 0.001
6148 reflections	Δρ_max_ = 0.63 e Å^−^^3^
406 parameters	Δρ_min_ = −0.45 e Å^−^^3^

### Special details

**Table:**

Geometry. All esds (except the esd in the dihedral angle between two l.s. planes) are estimated using the full covariance matrix. The cell esds are taken into account individually in the estimation of esds in distances, angles and torsion angles; correlations between esds in cell parameters are only used when they are defined by crystal symmetry. An approximate (isotropic) treatment of cell esds is used for estimating esds involving l.s. planes.

### Fractional atomic coordinates and isotropic or equivalent isotropic displacement parameters (Å^2^)

**Table:**

	*x*	*y*	*z*	*U*_iso_*/*U*_eq_	
Ni1	0.47434 (2)	0.39867 (5)	0.07544 (2)	0.01087 (9)	
K1	0.17619 (4)	0.53183 (9)	0.11705 (4)	0.01892 (16)	
K2	0.19572 (3)	0.36084 (9)	−0.07023 (4)	0.01670 (15)	
K3	0.27474 (3)	0.87045 (9)	0.01255 (4)	0.01761 (15)	
K4	0.37676 (4)	0.05611 (9)	−0.14504 (4)	0.02025 (16)	
O1	0.41934 (10)	0.2347 (3)	0.01663 (13)	0.0155 (5)	
O2	0.55123 (10)	0.2576 (3)	0.08187 (12)	0.0138 (4)	
O3	0.66101 (11)	0.2786 (3)	0.11647 (14)	0.0178 (5)	
O4	0.63860 (11)	0.6017 (3)	0.18521 (13)	0.0175 (5)	
O5	0.43257 (10)	0.6088 (3)	0.23723 (12)	0.0144 (4)	
O6	0.28491 (10)	0.5095 (3)	0.04127 (13)	0.0144 (5)	
O7	0.31296 (10)	0.1927 (3)	−0.03135 (13)	0.0145 (4)	
O8	0.23728 (13)	0.4224 (4)	0.26454 (15)	0.0296 (6)	
H8O	0.234956	0.311841	0.266821	0.044*	
H8P	0.220280	0.460928	0.303719	0.044*	
O9	0.22781 (12)	0.4456 (3)	−0.21414 (15)	0.0260 (6)	
H9P	0.270092	0.459040	−0.210682	0.039*	
H9O	0.209842	0.522979	−0.245902	0.039*	
O10	0.32908 (13)	1.1134 (4)	0.14694 (15)	0.0306 (6)	
H10O	0.287037	1.137097	0.135669	0.046*	
H10P	0.338827	1.126557	0.197749	0.046*	
N1	0.52942 (12)	0.5586 (3)	0.13120 (14)	0.0113 (5)	
N2	0.51068 (13)	0.7242 (3)	0.15852 (15)	0.0124 (5)	
N3	0.39268 (13)	0.6979 (3)	0.10530 (15)	0.0131 (5)	
N4	0.39871 (13)	0.5297 (3)	0.07165 (15)	0.0122 (5)	
C1	0.60452 (15)	0.3371 (4)	0.11225 (18)	0.0137 (6)	
C2	0.59253 (16)	0.5163 (4)	0.14705 (18)	0.0146 (6)	
C3	0.45459 (15)	0.7945 (4)	0.10429 (19)	0.0140 (6)	
H3A	0.447429	0.917052	0.118117	0.017*	
H3B	0.466161	0.792495	0.050662	0.017*	
C4	0.49161 (15)	0.7092 (4)	0.23624 (18)	0.0140 (6)	
H4A	0.528350	0.654533	0.271547	0.017*	
H4B	0.484584	0.826836	0.256513	0.017*	
C5	0.37883 (16)	0.6825 (4)	0.18499 (18)	0.0155 (6)	
H5A	0.368739	0.799144	0.204239	0.019*	
H5B	0.338951	0.609318	0.185349	0.019*	
C6	0.34231 (15)	0.4539 (4)	0.04236 (17)	0.0117 (6)	
C7	0.35790 (15)	0.2774 (4)	0.00678 (17)	0.0122 (6)	
Ni1B	0.01223 (2)	0.08795 (5)	0.09162 (2)	0.01243 (9)	
O1B	0.08679 (11)	0.2257 (3)	0.07813 (13)	0.0166 (5)	
O2B	−0.04769 (11)	0.2677 (3)	0.05952 (13)	0.0156 (5)	
O3B	−0.15683 (11)	0.3148 (3)	0.03336 (14)	0.0184 (5)	
O4B	−0.17542 (11)	−0.0118 (3)	0.10065 (13)	0.0164 (5)	
O5B	0.00334 (11)	−0.1490 (3)	0.25474 (13)	0.0170 (5)	
O6B	0.18685 (11)	−0.1330 (3)	0.14427 (14)	0.0185 (5)	
O7B	0.19555 (11)	0.1959 (3)	0.07804 (14)	0.0200 (5)	
N1B	−0.06184 (13)	−0.0419 (3)	0.10402 (15)	0.0133 (5)	
N2B	−0.06258 (13)	−0.2169 (3)	0.13205 (15)	0.0134 (5)	
N3B	0.05905 (13)	−0.2534 (3)	0.14984 (15)	0.0135 (5)	
N4B	0.07292 (13)	−0.0845 (3)	0.12181 (15)	0.0140 (5)	
C1B	−0.10848 (16)	0.2241 (4)	0.05852 (18)	0.0144 (6)	
C2B	−0.11930 (16)	0.0406 (4)	0.09058 (17)	0.0137 (6)	
C3B	−0.00599 (15)	−0.3127 (4)	0.10940 (19)	0.0146 (6)	
H3B1	−0.006462	−0.300402	0.052383	0.017*	
H3B2	−0.011318	−0.437434	0.120686	0.017*	
C4B	−0.05815 (16)	−0.2189 (4)	0.21681 (18)	0.0152 (6)	
H4B1	−0.095254	−0.150425	0.232299	0.018*	
H4B2	−0.062785	−0.339827	0.234578	0.018*	
C5B	0.05751 (16)	−0.2475 (4)	0.23366 (19)	0.0158 (6)	
H5B1	0.054706	−0.367518	0.253325	0.019*	
H5B2	0.099552	−0.196407	0.259598	0.019*	
C6B	0.13592 (16)	−0.0447 (4)	0.12181 (18)	0.0154 (6)	
C7B	0.14124 (15)	0.1389 (4)	0.08983 (18)	0.0143 (6)	

### Atomic displacement parameters (Å^2^)

**Table:**

	*U*^11^	*U*^22^	*U*^33^	*U*^12^	*U*^13^	*U*^23^
Ni1	0.0117 (2)	0.00866 (18)	0.01215 (19)	0.00042 (14)	0.00137 (14)	−0.00210 (15)
K1	0.0225 (4)	0.0128 (3)	0.0229 (4)	0.0021 (3)	0.0082 (3)	0.0018 (3)
K2	0.0181 (4)	0.0124 (3)	0.0182 (3)	0.0002 (3)	−0.0022 (3)	−0.0009 (3)
K3	0.0162 (3)	0.0130 (3)	0.0229 (4)	−0.0001 (3)	0.0006 (3)	0.0043 (3)
K4	0.0253 (4)	0.0118 (3)	0.0265 (4)	−0.0019 (3)	0.0134 (3)	−0.0026 (3)
O1	0.0140 (11)	0.0129 (11)	0.0195 (12)	−0.0001 (9)	0.0021 (9)	−0.0028 (9)
O2	0.0149 (11)	0.0116 (10)	0.0149 (11)	0.0018 (8)	0.0020 (8)	−0.0011 (9)
O3	0.0124 (11)	0.0158 (11)	0.0253 (13)	0.0022 (9)	0.0030 (9)	−0.0007 (10)
O4	0.0151 (11)	0.0154 (11)	0.0207 (12)	−0.0003 (9)	−0.0016 (9)	−0.0013 (9)
O5	0.0159 (11)	0.0146 (11)	0.0135 (11)	−0.0009 (9)	0.0044 (8)	0.0014 (9)
O6	0.0135 (11)	0.0127 (11)	0.0168 (11)	0.0013 (9)	0.0016 (9)	−0.0016 (9)
O7	0.0144 (11)	0.0138 (11)	0.0148 (11)	−0.0021 (9)	0.0005 (8)	−0.0015 (9)
O8	0.0368 (16)	0.0286 (14)	0.0253 (14)	0.0059 (12)	0.0103 (11)	0.0025 (11)
O9	0.0159 (13)	0.0345 (15)	0.0268 (14)	−0.0018 (11)	0.0006 (10)	0.0045 (11)
O10	0.0259 (14)	0.0390 (16)	0.0269 (14)	0.0052 (12)	0.0038 (11)	0.0047 (12)
N1	0.0137 (13)	0.0095 (12)	0.0107 (12)	0.0008 (10)	0.0022 (10)	−0.0013 (10)
N2	0.0151 (13)	0.0104 (12)	0.0118 (12)	0.0006 (10)	0.0018 (10)	−0.0017 (10)
N3	0.0177 (14)	0.0090 (12)	0.0125 (13)	0.0001 (10)	0.0021 (10)	−0.0040 (10)
N4	0.0176 (14)	0.0075 (12)	0.0116 (12)	0.0018 (10)	0.0024 (10)	−0.0028 (10)
C1	0.0176 (16)	0.0137 (15)	0.0105 (14)	−0.0010 (12)	0.0042 (12)	0.0031 (12)
C2	0.0179 (17)	0.0124 (15)	0.0141 (15)	−0.0020 (12)	0.0039 (12)	0.0019 (12)
C3	0.0158 (16)	0.0091 (14)	0.0164 (15)	0.0002 (12)	0.0002 (12)	−0.0002 (12)
C4	0.0174 (16)	0.0141 (15)	0.0106 (14)	0.0009 (12)	0.0022 (12)	−0.0019 (12)
C5	0.0177 (16)	0.0152 (15)	0.0139 (15)	−0.0005 (12)	0.0036 (12)	−0.0020 (12)
C6	0.0149 (15)	0.0110 (14)	0.0092 (14)	−0.0001 (12)	0.0023 (11)	0.0008 (11)
C7	0.0165 (16)	0.0098 (14)	0.0105 (14)	−0.0003 (11)	0.0026 (11)	0.0020 (11)
Ni1B	0.0128 (2)	0.00920 (19)	0.0149 (2)	−0.00029 (15)	0.00073 (15)	0.00233 (15)
O1B	0.0159 (12)	0.0116 (11)	0.0224 (12)	−0.0001 (9)	0.0028 (9)	0.0032 (9)
O2B	0.0159 (12)	0.0122 (11)	0.0179 (11)	−0.0007 (9)	−0.0001 (9)	0.0019 (9)
O3B	0.0165 (12)	0.0134 (11)	0.0246 (13)	0.0028 (9)	0.0003 (9)	0.0034 (10)
O4B	0.0149 (12)	0.0140 (11)	0.0203 (12)	0.0005 (9)	0.0021 (9)	0.0004 (9)
O5B	0.0202 (12)	0.0165 (12)	0.0138 (11)	0.0000 (9)	0.0007 (9)	−0.0009 (9)
O6B	0.0139 (11)	0.0157 (11)	0.0251 (12)	0.0009 (9)	0.0004 (9)	−0.0003 (10)
O7B	0.0164 (12)	0.0168 (12)	0.0277 (13)	−0.0025 (9)	0.0067 (10)	0.0025 (10)
N1B	0.0168 (14)	0.0075 (12)	0.0152 (13)	−0.0003 (10)	0.0009 (10)	0.0000 (10)
N2B	0.0158 (13)	0.0095 (12)	0.0149 (13)	0.0000 (10)	0.0021 (10)	0.0021 (10)
N3B	0.0154 (13)	0.0094 (12)	0.0154 (13)	−0.0013 (10)	0.0014 (10)	0.0048 (10)
N4B	0.0146 (13)	0.0101 (12)	0.0170 (13)	−0.0006 (10)	0.0018 (10)	0.0021 (10)
C1B	0.0197 (17)	0.0128 (15)	0.0109 (14)	−0.0019 (12)	0.0023 (12)	−0.0021 (12)
C2B	0.0188 (17)	0.0114 (14)	0.0104 (14)	−0.0001 (12)	0.0004 (12)	−0.0018 (11)
C3B	0.0133 (15)	0.0122 (15)	0.0176 (16)	−0.0013 (12)	0.0002 (12)	−0.0008 (12)
C4B	0.0157 (16)	0.0129 (15)	0.0165 (15)	−0.0008 (12)	0.0009 (12)	0.0037 (12)
C5B	0.0177 (17)	0.0127 (15)	0.0168 (16)	0.0014 (12)	0.0014 (12)	0.0041 (12)
C6B	0.0170 (16)	0.0154 (16)	0.0137 (15)	0.0001 (12)	0.0017 (12)	−0.0035 (12)
C7B	0.0163 (16)	0.0144 (15)	0.0122 (15)	−0.0005 (12)	0.0017 (12)	−0.0007 (12)

### Geometric parameters (Å, º)

**Table:**

Ni1—N4	1.836 (3)	O5—C4	1.433 (4)
Ni1—N1	1.844 (3)	O5—C5	1.434 (4)
Ni1—O1	1.887 (2)	O6—C6	1.244 (4)
Ni1—O2	1.897 (2)	O7—C7	1.236 (4)
K1—O6B^i^	2.628 (2)	O8—H8O	0.8546
K1—O7B	2.717 (2)	O8—H8P	0.8575
K1—O6	2.737 (2)	O9—H9P	0.8615
K1—O8	2.805 (3)	O9—H9O	0.8567
K1—O3B^ii^	2.834 (2)	O10—H10O	0.8704
K1—O1B	2.998 (2)	O10—H10P	0.8792
K1—N3B^i^	3.025 (3)	N1—C2	1.317 (4)
K1—C7B	3.130 (3)	N1—N2	1.431 (3)
K1—C6B^i^	3.368 (3)	N2—C4	1.457 (4)
K1—K2	3.5736 (10)	N2—C3	1.474 (4)
K1—K3	3.8927 (10)	N3—N4	1.433 (3)
K2—O6	2.708 (2)	N3—C5	1.452 (4)
K2—O7	2.717 (2)	N3—C3	1.467 (4)
K2—O3B^ii^	2.725 (2)	N4—C6	1.324 (4)
K2—O9	2.743 (3)	C1—C2	1.540 (4)
K2—O4B^iii^	2.760 (2)	C3—H3A	0.9900
K2—O7B	2.864 (2)	C3—H3B	0.9900
K2—N2B^iii^	2.984 (3)	C4—H4A	0.9900
K2—C6	3.401 (3)	C4—H4B	0.9900
K2—C7	3.443 (3)	C5—H5A	0.9900
K2—C2B^iii^	3.458 (3)	C5—H5B	0.9900
K2—K3^iv^	4.2728 (10)	C6—C7	1.544 (4)
K3—O7^i^	2.742 (2)	Ni1B—N4B	1.839 (3)
K3—O3B^ii^	2.810 (2)	Ni1B—N1B	1.849 (3)
K3—O4B^ii^	2.826 (2)	Ni1B—O2B	1.877 (2)
K3—O6	2.827 (2)	Ni1B—O1B	1.895 (2)
K3—O3^v^	2.976 (2)	O1B—C7B	1.287 (4)
K3—N3	3.003 (3)	O2B—C1B	1.281 (4)
K3—O10	3.066 (3)	O3B—C1B	1.235 (4)
K3—O6B^i^	3.095 (2)	O4B—C2B	1.249 (4)
K3—O7B^i^	3.271 (2)	O5B—C5B	1.430 (4)
K3—C2B^ii^	3.475 (3)	O5B—C4B	1.434 (4)
K3—C6	3.501 (3)	O6B—C6B	1.255 (4)
K3—C1B^ii^	3.512 (3)	O7B—C7B	1.235 (4)
K3—H10O	2.9446	N1B—C2B	1.323 (4)
K4—O7	2.721 (2)	N1B—N2B	1.434 (3)
K4—O4^v^	2.733 (2)	N2B—C4B	1.457 (4)
K4—O3^vi^	2.756 (2)	N2B—C3B	1.469 (4)
K4—O5^vii^	2.779 (2)	N3B—N4B	1.431 (3)
K4—N2^v^	2.887 (3)	N3B—C5B	1.456 (4)
K4—O2^vi^	2.955 (2)	N3B—C3B	1.480 (4)
K4—O8^vii^	3.047 (3)	N4B—C6B	1.320 (4)
K4—C1^vi^	3.095 (3)	C1B—C2B	1.546 (4)
K4—O1	3.127 (2)	C3B—H3B1	0.9900
K4—C7	3.201 (3)	C3B—H3B2	0.9900
K4—C2^v^	3.354 (3)	C4B—H4B1	0.9900
K4—C5^vii^	3.474 (3)	C4B—H4B2	0.9900
O1—C7	1.282 (4)	C5B—H5B1	0.9900
O2—C1	1.291 (4)	C5B—H5B2	0.9900
O3—C1	1.229 (4)	C6B—C7B	1.528 (4)
O4—C2	1.254 (4)		
			
N4—Ni1—N1	95.53 (11)	N2^v^—K4—O1	72.10 (7)
N4—Ni1—O1	85.30 (10)	O2^vi^—K4—O1	88.24 (6)
N1—Ni1—O1	178.66 (11)	O8^vii^—K4—O1	123.68 (7)
N4—Ni1—O2	177.92 (11)	C1^vi^—K4—O1	104.79 (7)
N1—Ni1—O2	85.18 (10)	O7—K4—C7	22.25 (7)
O1—Ni1—O2	94.02 (9)	O4^v^—K4—C7	70.87 (7)
O6B^i^—K1—O7B	165.68 (7)	O3^vi^—K4—C7	106.35 (7)
O6B^i^—K1—O6	95.50 (7)	O5^vii^—K4—C7	162.77 (8)
O7B—K1—O6	70.41 (7)	N2^v^—K4—C7	86.82 (8)
O6B^i^—K1—O8	96.75 (8)	O2^vi^—K4—C7	104.10 (7)
O7B—K1—O8	83.03 (8)	O8^vii^—K4—C7	100.36 (8)
O6—K1—O8	97.64 (7)	C1^vi^—K4—C7	113.38 (8)
O6B^i^—K1—O3B^ii^	75.65 (7)	O1—K4—C7	23.34 (7)
O7B—K1—O3B^ii^	100.07 (7)	O7—K4—C2^v^	74.75 (7)
O6—K1—O3B^ii^	66.54 (7)	O4^v^—K4—C2^v^	20.73 (7)
O8—K1—O3B^ii^	161.16 (8)	O3^vi^—K4—C2^v^	167.98 (8)
O6B^i^—K1—O1B	147.71 (7)	O5^vii^—K4—C2^v^	110.16 (7)
O7B—K1—O1B	45.58 (6)	N2^v^—K4—C2^v^	43.27 (7)
O6—K1—O1B	110.52 (7)	O2^vi^—K4—C2^v^	136.36 (7)
O8—K1—O1B	98.30 (7)	O8^vii^—K4—C2^v^	95.92 (8)
O3B^ii^—K1—O1B	96.98 (7)	C1^vi^—K4—C2^v^	160.86 (8)
O6B^i^—K1—N3B^i^	58.41 (7)	O1—K4—C2^v^	63.19 (7)
O7B—K1—N3B^i^	135.57 (7)	C7—K4—C2^v^	61.97 (8)
O6—K1—N3B^i^	147.07 (7)	O7—K4—C5^vii^	151.88 (7)
O8—K1—N3B^i^	104.66 (8)	O4^v^—K4—C5^vii^	108.05 (7)
O3B^ii^—K1—N3B^i^	86.25 (7)	O3^vi^—K4—C5^vii^	72.21 (7)
O1B—K1—N3B^i^	90.10 (7)	O5^vii^—K4—C5^vii^	23.29 (7)
O6B^i^—K1—C7B	171.25 (8)	N2^v^—K4—C5^vii^	97.79 (7)
O7B—K1—C7B	23.02 (7)	O2^vi^—K4—C5^vii^	79.33 (7)
O6—K1—C7B	92.84 (8)	O8^vii^—K4—C5^vii^	73.58 (7)
O8—K1—C7B	84.73 (8)	C1^vi^—K4—C5^vii^	67.75 (8)
O3B^ii^—K1—C7B	105.44 (8)	O1—K4—C5^vii^	162.73 (7)
O1B—K1—C7B	24.11 (7)	C7—K4—C5^vii^	173.84 (8)
N3B^i^—K1—C7B	112.86 (8)	C2^v^—K4—C5^vii^	119.08 (8)
O6B^i^—K1—C6B^i^	19.63 (7)	C7—O1—Ni1	113.14 (19)
O7B—K1—C6B^i^	166.68 (8)	C7—O1—K4	81.58 (17)
O6—K1—C6B^i^	106.83 (7)	Ni1—O1—K4	147.60 (10)
O8—K1—C6B^i^	110.29 (8)	C1—O2—Ni1	113.09 (19)
O3B^ii^—K1—C6B^i^	67.44 (7)	C1—O2—K4^vi^	83.85 (17)
O1B—K1—C6B^i^	128.70 (7)	Ni1—O2—K4^vi^	148.49 (10)
N3B^i^—K1—C6B^i^	42.60 (7)	C1—O3—K4^vi^	94.03 (19)
C7B—K1—C6B^i^	152.81 (8)	C1—O3—K3^v^	127.3 (2)
O6B^i^—K1—K2	120.51 (6)	K4^vi^—O3—K3^v^	86.44 (6)
O7B—K1—K2	52.02 (5)	C2—O4—K4^v^	108.80 (19)
O6—K1—K2	48.63 (5)	C4—O5—C5	110.2 (2)
O8—K1—K2	128.47 (6)	C4—O5—K4^viii^	133.50 (17)
O3B^ii^—K1—K2	48.67 (5)	C5—O5—K4^viii^	106.70 (16)
O1B—K1—K2	69.47 (5)	C6—O6—K2	113.52 (19)
N3B^i^—K1—K2	124.43 (6)	C6—O6—K1	147.0 (2)
C7B—K1—K2	63.86 (6)	K2—O6—K1	82.02 (6)
C6B^i^—K1—K2	116.01 (6)	C6—O6—K3	112.75 (18)
O6B^i^—K1—K3	52.41 (5)	K2—O6—K3	105.41 (7)
O7B—K1—K3	114.82 (5)	K1—O6—K3	88.76 (6)
O6—K1—K3	46.56 (5)	C7—O7—K2	116.01 (19)
O8—K1—K3	115.66 (6)	C7—O7—K4	101.30 (18)
O3B^ii^—K1—K3	46.14 (5)	K2—O7—K4	119.99 (8)
O1B—K1—K3	139.50 (5)	C7—O7—K3^iv^	123.27 (19)
N3B^i^—K1—K3	101.21 (5)	K2—O7—K3^iv^	103.04 (7)
C7B—K1—K3	134.40 (6)	K4—O7—K3^iv^	91.98 (7)
C6B^i^—K1—K3	60.40 (6)	K1—O8—K4^viii^	135.32 (10)
K2—K1—K3	72.16 (2)	K1—O8—H8O	108.7
O6—K2—O7	63.15 (6)	K4^viii^—O8—H8O	94.8
O6—K2—O3B^ii^	68.50 (7)	K1—O8—H8P	116.0
O7—K2—O3B^ii^	130.88 (7)	K4^viii^—O8—H8P	91.6
O6—K2—O9	108.86 (7)	H8O—O8—H8P	106.1
O7—K2—O9	91.30 (7)	K2—O9—H9P	109.4
O3B^ii^—K2—O9	96.29 (8)	K2—O9—H9O	127.8
O6—K2—O4B^iii^	127.95 (7)	H9P—O9—H9O	107.0
O7—K2—O4B^iii^	71.65 (7)	K3—O10—H10O	73.8
O3B^ii^—K2—O4B^iii^	153.83 (7)	K3—O10—H10P	146.5
O9—K2—O4B^iii^	96.17 (8)	H10O—O10—H10P	105.9
O6—K2—O7B	68.65 (7)	C2—N1—N2	116.8 (3)
O7—K2—O7B	71.37 (7)	C2—N1—Ni1	116.5 (2)
O3B^ii^—K2—O7B	99.16 (7)	N2—N1—Ni1	126.66 (19)
O9—K2—O7B	161.85 (8)	N1—N2—C4	110.7 (2)
O4B^iii^—K2—O7B	73.70 (7)	N1—N2—C3	109.7 (2)
O6—K2—N2B^iii^	153.59 (7)	C4—N2—C3	109.4 (2)
O7—K2—N2B^iii^	129.35 (7)	N1—N2—K4^v^	104.06 (16)
O3B^ii^—K2—N2B^iii^	98.31 (7)	C4—N2—K4^v^	116.19 (18)
O9—K2—N2B^iii^	94.92 (7)	C3—N2—K4^v^	106.62 (17)
O4B^iii^—K2—N2B^iii^	57.71 (7)	N4—N3—C5	110.6 (2)
O7B—K2—N2B^iii^	92.22 (7)	N4—N3—C3	109.3 (2)
O6—K2—C6	19.59 (7)	C5—N3—C3	109.7 (2)
O7—K2—C6	44.71 (7)	N4—N3—K3	106.96 (16)
O3B^ii^—K2—C6	86.20 (7)	C5—N3—K3	107.10 (18)
O9—K2—C6	99.55 (7)	C3—N3—K3	113.13 (18)
O4B^iii^—K2—C6	114.21 (7)	C6—N4—N3	115.7 (2)
O7B—K2—C6	72.17 (7)	C6—N4—Ni1	116.7 (2)
N2B^iii^—K2—C6	164.29 (7)	N3—N4—Ni1	127.1 (2)
O6—K2—C7	45.08 (7)	O3—C1—O2	125.2 (3)
O7—K2—C7	18.82 (7)	O3—C1—C2	120.4 (3)
O3B^ii^—K2—C7	112.08 (7)	O2—C1—C2	114.4 (3)
O9—K2—C7	93.17 (7)	O3—C1—K4^vi^	62.64 (17)
O4B^iii^—K2—C7	90.08 (7)	O2—C1—K4^vi^	71.65 (16)
O7B—K2—C7	72.22 (7)	C2—C1—K4^vi^	145.90 (19)
N2B^iii^—K2—C7	147.43 (7)	O4—C2—N1	127.9 (3)
C6—K2—C7	26.07 (7)	O4—C2—C1	121.8 (3)
O6—K2—C2B^iii^	134.22 (7)	N1—C2—C1	110.2 (3)
O7—K2—C2B^iii^	88.06 (7)	O4—C2—K4^v^	50.47 (16)
O3B^ii^—K2—C2B^iii^	134.59 (7)	N1—C2—K4^v^	86.15 (18)
O9—K2—C2B^iii^	106.44 (8)	C1—C2—K4^v^	146.74 (19)
O4B^iii^—K2—C2B^iii^	19.29 (7)	N3—C3—N2	113.3 (2)
O7B—K2—C2B^iii^	68.67 (7)	N3—C3—H3A	108.9
N2B^iii^—K2—C2B^iii^	42.10 (7)	N2—C3—H3A	108.9
C6—K2—C2B^iii^	126.35 (7)	N3—C3—H3B	108.9
C7—K2—C2B^iii^	105.43 (7)	N2—C3—H3B	108.9
O6—K2—K1	49.34 (5)	H3A—C3—H3B	107.7
O7—K2—K1	99.18 (5)	O5—C4—N2	112.9 (2)
O3B^ii^—K2—K1	51.36 (5)	O5—C4—H4A	109.0
O9—K2—K1	143.89 (6)	N2—C4—H4A	109.0
O4B^iii^—K2—K1	119.94 (5)	O5—C4—H4B	109.0
O7B—K2—K1	48.41 (5)	N2—C4—H4B	109.0
N2B^iii^—K2—K1	104.38 (6)	H4A—C4—H4B	107.8
C6—K2—K1	66.77 (5)	O5—C5—N3	113.3 (2)
C7—K2—K1	86.75 (5)	O5—C5—K4^viii^	50.01 (13)
C2B^iii^—K2—K1	108.36 (5)	N3—C5—K4^viii^	151.1 (2)
O6—K2—K3^iv^	87.29 (5)	O5—C5—H5A	108.9
O7—K2—K3^iv^	38.69 (5)	N3—C5—H5A	108.9
O3B^ii^—K2—K3^iv^	147.21 (6)	K4^viii^—C5—H5A	99.6
O9—K2—K3^iv^	112.80 (6)	O5—C5—H5B	108.9
O4B^iii^—K2—K3^iv^	40.67 (5)	N3—C5—H5B	108.9
O7B—K2—K3^iv^	49.92 (5)	K4^viii^—C5—H5B	65.4
N2B^iii^—K2—K3^iv^	94.10 (5)	H5A—C5—H5B	107.7
C6—K2—K3^iv^	74.71 (5)	O6—C6—N4	128.1 (3)
C7—K2—K3^iv^	53.87 (5)	O6—C6—C7	123.0 (3)
C2B^iii^—K2—K3^iv^	52.13 (5)	N4—C6—C7	108.9 (3)
K1—K2—K3^iv^	96.17 (2)	O6—C6—K2	46.89 (15)
O7^i^—K3—O3B^ii^	130.32 (7)	N4—C6—K2	162.9 (2)
O7^i^—K3—O4B^ii^	70.29 (6)	C7—C6—K2	78.47 (16)
O3B^ii^—K3—O4B^ii^	60.07 (7)	O6—C6—K3	48.13 (15)
O7^i^—K3—O6	157.32 (7)	N4—C6—K3	87.33 (18)
O3B^ii^—K3—O6	65.69 (6)	C7—C6—K3	147.92 (19)
O4B^ii^—K3—O6	121.88 (7)	K2—C6—K3	79.28 (7)
O7^i^—K3—O3^v^	88.18 (7)	O7—C7—O1	124.8 (3)
O3B^ii^—K3—O3^v^	92.43 (7)	O7—C7—C6	119.9 (3)
O4B^ii^—K3—O3^v^	88.69 (7)	O1—C7—C6	115.2 (3)
O6—K3—O3^v^	73.94 (6)	O7—C7—K4	56.45 (16)
O7^i^—K3—N3	108.15 (7)	O1—C7—K4	75.08 (16)
O3B^ii^—K3—N3	120.86 (7)	C6—C7—K4	148.43 (19)
O4B^ii^—K3—N3	168.61 (7)	O7—C7—K2	45.16 (15)
O6—K3—N3	55.78 (7)	O1—C7—K2	164.7 (2)
O3^v^—K3—N3	79.96 (7)	C6—C7—K2	75.46 (16)
O7^i^—K3—O10	64.62 (7)	K4—C7—K2	90.18 (8)
O3B^ii^—K3—O10	136.54 (7)	N4B—Ni1B—N1B	95.93 (11)
O4B^ii^—K3—O10	115.82 (7)	N4B—Ni1B—O2B	178.25 (11)
O6—K3—O10	117.23 (7)	N1B—Ni1B—O2B	85.75 (10)
O3^v^—K3—O10	130.90 (7)	N4B—Ni1B—O1B	85.46 (10)
N3—K3—O10	71.96 (7)	N1B—Ni1B—O1B	178.61 (11)
O7^i^—K3—O6B^i^	115.43 (7)	O2B—Ni1B—O1B	92.86 (9)
O3B^ii^—K3—O6B^i^	69.03 (7)	C7B—O1B—Ni1B	112.2 (2)
O4B^ii^—K3—O6B^i^	94.66 (6)	C7B—O1B—K1	83.73 (17)
O6—K3—O6B^i^	84.13 (6)	Ni1B—O1B—K1	152.27 (11)
O3^v^—K3—O6B^i^	155.90 (7)	C1B—O2B—Ni1B	113.4 (2)
N3—K3—O6B^i^	96.08 (7)	C1B—O3B—K2^ii^	132.9 (2)
O10—K3—O6B^i^	68.32 (7)	C1B—O3B—K3^ii^	114.8 (2)
O7^i^—K3—O7B^i^	64.91 (6)	K2^ii^—O3B—K3^ii^	105.44 (8)
O3B^ii^—K3—O7B^i^	92.46 (6)	C1B—O3B—K1^ii^	124.0 (2)
O4B^ii^—K3—O7B^i^	66.72 (6)	K2^ii^—O3B—K1^ii^	79.97 (6)
O6—K3—O7B^i^	136.08 (6)	K3^ii^—O3B—K1^ii^	87.20 (7)
O3^v^—K3—O7B^i^	147.96 (7)	C2B—O4B—K2^iii^	113.82 (19)
N3—K3—O7B^i^	123.44 (7)	C2B—O4B—K3^ii^	110.98 (19)
O10—K3—O7B^i^	53.97 (6)	K2^iii^—O4B—K3^ii^	99.81 (7)
O6B^i^—K3—O7B^i^	52.04 (6)	C5B—O5B—C4B	109.9 (2)
O7^i^—K3—C2B^ii^	87.34 (7)	C6B—O6B—K1^iv^	115.7 (2)
O3B^ii^—K3—C2B^ii^	43.53 (7)	C6B—O6B—K3^iv^	107.9 (2)
O4B^ii^—K3—C2B^ii^	19.60 (7)	K1^iv^—O6B—K3^iv^	85.30 (7)
O6—K3—C2B^ii^	108.83 (7)	C7B—O7B—K1	97.60 (19)
O3^v^—K3—C2B^ii^	99.07 (7)	C7B—O7B—K2	115.0 (2)
N3—K3—C2B^ii^	164.39 (7)	K1—O7B—K2	79.58 (6)
O10—K3—C2B^ii^	118.22 (7)	C7B—O7B—K3^iv^	106.4 (2)
O6B^i^—K3—C2B^ii^	78.38 (7)	K1—O7B—K3^iv^	155.85 (9)
O7B^i^—K3—C2B^ii^	64.41 (7)	K2—O7B—K3^iv^	88.02 (6)
O7^i^—K3—C6	138.54 (7)	C2B—N1B—N2B	117.3 (3)
O3B^ii^—K3—C6	83.02 (7)	C2B—N1B—Ni1B	115.9 (2)
O4B^ii^—K3—C6	133.08 (7)	N2B—N1B—Ni1B	126.6 (2)
O6—K3—C6	19.12 (6)	N1B—N2B—C4B	110.4 (2)
O3^v^—K3—C6	63.31 (7)	N1B—N2B—C3B	109.6 (2)
N3—K3—C6	41.19 (7)	C4B—N2B—C3B	109.0 (2)
O10—K3—C6	110.86 (7)	N1B—N2B—K2^iii^	106.33 (17)
O6B^i^—K3—C6	98.09 (7)	C4B—N2B—K2^iii^	106.21 (18)
O7B^i^—K3—C6	148.73 (7)	C3B—N2B—K2^iii^	115.23 (18)
C2B^ii^—K3—C6	124.54 (7)	N4B—N3B—C5B	110.0 (2)
O7^i^—K3—C1B^ii^	112.89 (7)	N4B—N3B—C3B	109.1 (2)
O3B^ii^—K3—C1B^ii^	18.63 (7)	C5B—N3B—C3B	109.6 (2)
O4B^ii^—K3—C1B^ii^	43.75 (7)	N4B—N3B—K1^iv^	103.54 (16)
O6—K3—C1B^ii^	84.28 (7)	C5B—N3B—K1^iv^	109.54 (18)
O3^v^—K3—C1B^ii^	99.41 (7)	C3B—N3B—K1^iv^	114.86 (18)
N3—K3—C1B^ii^	138.92 (7)	C6B—N4B—N3B	116.8 (3)
O10—K3—C1B^ii^	128.16 (7)	C6B—N4B—Ni1B	116.5 (2)
O6B^i^—K3—C1B^ii^	67.97 (7)	N3B—N4B—Ni1B	126.6 (2)
O7B^i^—K3—C1B^ii^	77.36 (7)	O3B—C1B—O2B	125.3 (3)
C2B^ii^—K3—C1B^ii^	25.56 (7)	O3B—C1B—C2B	119.7 (3)
C6—K3—C1B^ii^	101.59 (7)	O2B—C1B—C2B	115.0 (3)
O7^i^—K3—H10O	64.6	O3B—C1B—K3^ii^	46.60 (16)
O3B^ii^—K3—H10O	122.7	O2B—C1B—K3^ii^	160.3 (2)
O4B^ii^—K3—H10O	102.2	C2B—C1B—K3^ii^	75.88 (17)
O6—K3—H10O	124.2	O4B—C2B—N1B	128.7 (3)
O3^v^—K3—H10O	144.2	O4B—C2B—C1B	121.8 (3)
N3—K3—H10O	86.8	N1B—C2B—C1B	109.5 (3)
O10—K3—H10O	16.5	O4B—C2B—K2^iii^	46.89 (15)
O6B^i^—K3—H10O	58.1	N1B—C2B—K2^iii^	87.87 (18)
O7B^i^—K3—H10O	37.8	C1B—C2B—K2^iii^	150.1 (2)
C2B^ii^—K3—H10O	102.2	O4B—C2B—K3^ii^	49.41 (15)
C6—K3—H10O	122.8	N1B—C2B—K3^ii^	154.7 (2)
C1B^ii^—K3—H10O	112.1	C1B—C2B—K3^ii^	78.56 (17)
O7—K4—O4^v^	76.22 (7)	K2^iii^—C2B—K3^ii^	76.10 (7)
O7—K4—O3^vi^	93.28 (7)	N2B—C3B—N3B	113.8 (3)
O4^v^—K4—O3^vi^	157.43 (7)	N2B—C3B—H3B1	108.8
O7—K4—O5^vii^	174.39 (7)	N3B—C3B—H3B1	108.8
O4^v^—K4—O5^vii^	107.36 (7)	N2B—C3B—H3B2	108.8
O3^vi^—K4—O5^vii^	81.84 (7)	N3B—C3B—H3B2	108.8
O7—K4—N2^v^	107.43 (7)	H3B1—C3B—H3B2	107.7
O4^v^—K4—N2^v^	58.50 (7)	O5B—C4B—N2B	112.4 (3)
O3^vi^—K4—N2^v^	143.96 (7)	O5B—C4B—H4B1	109.1
O5^vii^—K4—N2^v^	78.18 (7)	N2B—C4B—H4B1	109.1
O7—K4—O2^vi^	108.20 (7)	O5B—C4B—H4B2	109.1
O4^v^—K4—O2^vi^	156.33 (7)	N2B—C4B—H4B2	109.1
O3^vi^—K4—O2^vi^	45.97 (6)	H4B1—C4B—H4B2	107.9
O5^vii^—K4—O2^vi^	70.34 (6)	O5B—C5B—N3B	113.5 (2)
N2^v^—K4—O2^vi^	98.69 (7)	O5B—C5B—H5B1	108.9
O7—K4—O8^vii^	80.97 (7)	N3B—C5B—H5B1	108.9
O4^v^—K4—O8^vii^	75.67 (7)	O5B—C5B—H5B2	108.9
O3^vi^—K4—O8^vii^	83.04 (7)	N3B—C5B—H5B2	108.9
O5^vii^—K4—O8^vii^	95.62 (7)	H5B1—C5B—H5B2	107.7
N2^v^—K4—O8^vii^	128.35 (8)	O6B—C6B—N4B	129.5 (3)
O2^vi^—K4—O8^vii^	127.72 (7)	O6B—C6B—C7B	121.0 (3)
O7—K4—C1^vi^	107.76 (8)	N4B—C6B—C7B	109.5 (3)
O4^v^—K4—C1^vi^	175.75 (8)	O6B—C6B—K1^iv^	44.70 (15)
O3^vi^—K4—C1^vi^	23.33 (7)	N4B—C6B—K1^iv^	90.89 (19)
O5^vii^—K4—C1^vi^	68.56 (7)	C7B—C6B—K1^iv^	149.0 (2)
N2^v^—K4—C1^vi^	120.66 (8)	O7B—C7B—O1B	124.2 (3)
O2^vi^—K4—C1^vi^	24.50 (7)	O7B—C7B—C6B	120.1 (3)
O8^vii^—K4—C1^vi^	103.22 (8)	O1B—C7B—C6B	115.7 (3)
O7—K4—O1	44.19 (6)	O7B—C7B—K1	59.38 (17)
O4^v^—K4—O1	79.06 (6)	O1B—C7B—K1	72.16 (17)
O3^vi^—K4—O1	107.45 (7)	C6B—C7B—K1	150.1 (2)
O5^vii^—K4—O1	140.07 (6)		
			
N4—Ni1—O1—C7	−5.2 (2)	K3—C6—C7—O1	121.4 (3)
O2—Ni1—O1—C7	172.8 (2)	O6—C6—C7—K4	80.9 (5)
N4—Ni1—O1—K4	106.9 (2)	N4—C6—C7—K4	−98.5 (4)
O2—Ni1—O1—K4	−75.08 (19)	K2—C6—C7—K4	65.4 (3)
N1—Ni1—O2—C1	−6.6 (2)	K3—C6—C7—K4	18.5 (7)
O1—Ni1—O2—C1	172.3 (2)	O6—C6—C7—K2	15.5 (3)
N1—Ni1—O2—K4^vi^	111.6 (2)	N4—C6—C7—K2	−163.9 (2)
O1—Ni1—O2—K4^vi^	−69.5 (2)	K3—C6—C7—K2	−46.9 (3)
N4—Ni1—N1—C2	−179.1 (2)	N4B—Ni1B—O1B—C7B	4.4 (2)
O2—Ni1—N1—C2	2.9 (2)	O2B—Ni1B—O1B—C7B	−175.1 (2)
N4—Ni1—N1—N2	0.5 (2)	N4B—Ni1B—O1B—K1	−117.2 (2)
O2—Ni1—N1—N2	−177.6 (2)	O2B—Ni1B—O1B—K1	63.2 (2)
C2—N1—N2—C4	−90.7 (3)	N1B—Ni1B—O2B—C1B	−1.5 (2)
Ni1—N1—N2—C4	89.7 (3)	O1B—Ni1B—O2B—C1B	178.5 (2)
C2—N1—N2—C3	148.5 (3)	N4B—Ni1B—N1B—C2B	177.3 (2)
Ni1—N1—N2—C3	−31.1 (3)	O2B—Ni1B—N1B—C2B	−3.2 (2)
C2—N1—N2—K4^v^	34.8 (3)	N4B—Ni1B—N1B—N2B	2.3 (3)
Ni1—N1—N2—K4^v^	−144.78 (16)	O2B—Ni1B—N1B—N2B	−178.2 (2)
C5—N3—N4—C6	83.1 (3)	C2B—N1B—N2B—C4B	−86.4 (3)
C3—N3—N4—C6	−156.0 (3)	Ni1B—N1B—N2B—C4B	88.5 (3)
K3—N3—N4—C6	−33.3 (3)	C2B—N1B—N2B—C3B	153.5 (3)
C5—N3—N4—Ni1	−88.9 (3)	Ni1B—N1B—N2B—C3B	−31.5 (3)
C3—N3—N4—Ni1	32.0 (3)	C2B—N1B—N2B—K2^iii^	28.4 (3)
K3—N3—N4—Ni1	154.80 (16)	Ni1B—N1B—N2B—K2^iii^	−156.67 (16)
N1—Ni1—N4—C6	−172.8 (2)	C5B—N3B—N4B—C6B	89.9 (3)
O1—Ni1—N4—C6	8.2 (2)	C3B—N3B—N4B—C6B	−149.9 (3)
N1—Ni1—N4—N3	−1.0 (2)	K1^iv^—N3B—N4B—C6B	−27.1 (3)
O1—Ni1—N4—N3	−179.9 (2)	C5B—N3B—N4B—Ni1B	−85.9 (3)
K4^vi^—O3—C1—O2	−36.9 (3)	C3B—N3B—N4B—Ni1B	34.3 (3)
K3^v^—O3—C1—O2	51.7 (4)	K1^iv^—N3B—N4B—Ni1B	157.07 (16)
K4^vi^—O3—C1—C2	141.0 (2)	N1B—Ni1B—N4B—C6B	−179.7 (2)
K3^v^—O3—C1—C2	−130.4 (2)	O1B—Ni1B—N4B—C6B	0.3 (2)
K3^v^—O3—C1—K4^vi^	88.59 (18)	N1B—Ni1B—N4B—N3B	−3.9 (3)
Ni1—O2—C1—O3	−173.4 (2)	O1B—Ni1B—N4B—N3B	176.1 (2)
K4^vi^—O2—C1—O3	34.2 (3)	K2^ii^—O3B—C1B—O2B	−57.3 (4)
Ni1—O2—C1—C2	8.5 (3)	K3^ii^—O3B—C1B—O2B	156.7 (2)
K4^vi^—O2—C1—C2	−143.9 (2)	K1^ii^—O3B—C1B—O2B	52.5 (4)
Ni1—O2—C1—K4^vi^	152.39 (15)	K2^ii^—O3B—C1B—C2B	124.0 (3)
K4^v^—O4—C2—N1	−41.1 (4)	K3^ii^—O3B—C1B—C2B	−22.0 (3)
K4^v^—O4—C2—C1	139.8 (2)	K1^ii^—O3B—C1B—C2B	−126.2 (2)
N2—N1—C2—O4	2.0 (5)	K2^ii^—O3B—C1B—K3^ii^	146.0 (3)
Ni1—N1—C2—O4	−178.4 (3)	K1^ii^—O3B—C1B—K3^ii^	−104.2 (2)
N2—N1—C2—C1	−178.8 (2)	Ni1B—O2B—C1B—O3B	−173.6 (3)
Ni1—N1—C2—C1	0.8 (3)	Ni1B—O2B—C1B—C2B	5.2 (3)
N2—N1—C2—K4^v^	−28.5 (2)	Ni1B—O2B—C1B—K3^ii^	−115.0 (6)
Ni1—N1—C2—K4^v^	151.09 (15)	K2^iii^—O4B—C2B—N1B	−35.3 (4)
O3—C1—C2—O4	−5.2 (5)	K3^ii^—O4B—C2B—N1B	−147.0 (3)
O2—C1—C2—O4	173.0 (3)	K2^iii^—O4B—C2B—C1B	144.7 (2)
K4^vi^—C1—C2—O4	79.9 (4)	K3^ii^—O4B—C2B—C1B	33.1 (3)
O3—C1—C2—N1	175.5 (3)	K3^ii^—O4B—C2B—K2^iii^	−111.64 (18)
O2—C1—C2—N1	−6.3 (4)	K2^iii^—O4B—C2B—K3^ii^	111.64 (18)
K4^vi^—C1—C2—N1	−99.3 (4)	N2B—N1B—C2B—O4B	1.8 (5)
O3—C1—C2—K4^v^	60.0 (5)	Ni1B—N1B—C2B—O4B	−173.7 (3)
O2—C1—C2—K4^v^	−121.8 (3)	N2B—N1B—C2B—C1B	−178.3 (2)
K4^vi^—C1—C2—K4^v^	145.1 (2)	Ni1B—N1B—C2B—C1B	6.2 (3)
N4—N3—C3—N2	−69.2 (3)	N2B—N1B—C2B—K2^iii^	−23.2 (2)
C5—N3—C3—N2	52.2 (3)	Ni1B—N1B—C2B—K2^iii^	161.31 (15)
K3—N3—C3—N2	171.75 (18)	N2B—N1B—C2B—K3^ii^	−73.4 (6)
N1—N2—C3—N3	69.0 (3)	Ni1B—N1B—C2B—K3^ii^	111.1 (5)
C4—N2—C3—N3	−52.6 (3)	O3B—C1B—C2B—O4B	−8.7 (4)
K4^v^—N2—C3—N3	−178.98 (19)	O2B—C1B—C2B—O4B	172.4 (3)
C5—O5—C4—N2	−57.2 (3)	K3^ii^—C1B—C2B—O4B	−25.0 (3)
K4^viii^—O5—C4—N2	162.24 (18)	O3B—C1B—C2B—N1B	171.3 (3)
N1—N2—C4—O5	−66.0 (3)	O2B—C1B—C2B—N1B	−7.5 (4)
C3—N2—C4—O5	54.9 (3)	K3^ii^—C1B—C2B—N1B	155.0 (2)
K4^v^—N2—C4—O5	175.62 (17)	O3B—C1B—C2B—K2^iii^	48.9 (5)
C4—O5—C5—N3	56.9 (3)	O2B—C1B—C2B—K2^iii^	−129.9 (3)
K4^viii^—O5—C5—N3	−151.9 (2)	K3^ii^—C1B—C2B—K2^iii^	32.6 (4)
C4—O5—C5—K4^viii^	−151.2 (2)	O3B—C1B—C2B—K3^ii^	16.3 (3)
N4—N3—C5—O5	66.4 (3)	O2B—C1B—C2B—K3^ii^	−162.6 (3)
C3—N3—C5—O5	−54.2 (3)	N1B—N2B—C3B—N3B	68.4 (3)
K3—N3—C5—O5	−177.35 (19)	C4B—N2B—C3B—N3B	−52.5 (3)
N4—N3—C5—K4^viii^	18.2 (5)	K2^iii^—N2B—C3B—N3B	−171.76 (18)
C3—N3—C5—K4^viii^	−102.4 (4)	N4B—N3B—C3B—N2B	−69.9 (3)
K3—N3—C5—K4^viii^	134.4 (3)	C5B—N3B—C3B—N2B	50.6 (3)
K2—O6—C6—N4	158.3 (3)	K1^iv^—N3B—C3B—N2B	174.42 (18)
K1—O6—C6—N4	−88.6 (5)	C5B—O5B—C4B—N2B	−59.4 (3)
K3—O6—C6—N4	38.5 (4)	N1B—N2B—C4B—O5B	−63.8 (3)
K2—O6—C6—C7	−21.0 (3)	C3B—N2B—C4B—O5B	56.6 (3)
K1—O6—C6—C7	92.1 (4)	K2^iii^—N2B—C4B—O5B	−178.71 (19)
K3—O6—C6—C7	−140.8 (2)	C4B—O5B—C5B—N3B	57.7 (3)
K1—O6—C6—K2	113.1 (4)	N4B—N3B—C5B—O5B	67.1 (3)
K3—O6—C6—K2	−119.79 (19)	C3B—N3B—C5B—O5B	−52.9 (3)
K2—O6—C6—K3	119.79 (19)	K1^iv^—N3B—C5B—O5B	−179.72 (19)
K1—O6—C6—K3	−127.1 (4)	K1^iv^—O6B—C6B—N4B	36.6 (4)
N3—N4—C6—O6	−0.9 (5)	K3^iv^—O6B—C6B—N4B	130.0 (3)
Ni1—N4—C6—O6	171.9 (2)	K1^iv^—O6B—C6B—C7B	−144.6 (2)
N3—N4—C6—C7	178.5 (2)	K3^iv^—O6B—C6B—C7B	−51.1 (3)
Ni1—N4—C6—C7	−8.7 (3)	K3^iv^—O6B—C6B—K1^iv^	93.42 (17)
N3—N4—C6—K2	65.3 (8)	N3B—N4B—C6B—O6B	−1.3 (5)
Ni1—N4—C6—K2	−121.9 (6)	Ni1B—N4B—C6B—O6B	174.9 (3)
N3—N4—C6—K3	26.8 (2)	N3B—N4B—C6B—C7B	179.7 (2)
Ni1—N4—C6—K3	−160.42 (15)	Ni1B—N4B—C6B—C7B	−4.0 (3)
K2—O7—C7—O1	−164.9 (2)	N3B—N4B—C6B—K1^iv^	23.5 (2)
K4—O7—C7—O1	−33.2 (3)	Ni1B—N4B—C6B—K1^iv^	−160.30 (15)
K3^iv^—O7—C7—O1	66.7 (4)	K1—O7B—C7B—O1B	33.6 (3)
K2—O7—C7—C6	11.3 (3)	K2—O7B—C7B—O1B	−48.4 (4)
K4—O7—C7—C6	142.9 (2)	K3^iv^—O7B—C7B—O1B	−144.0 (3)
K3^iv^—O7—C7—C6	−117.1 (2)	K1—O7B—C7B—C6B	−145.2 (2)
K2—O7—C7—K4	−131.67 (18)	K2—O7B—C7B—C6B	132.8 (2)
K3^iv^—O7—C7—K4	99.98 (18)	K3^iv^—O7B—C7B—C6B	37.2 (3)
K4—O7—C7—K2	131.67 (18)	K2—O7B—C7B—K1	−82.00 (13)
K3^iv^—O7—C7—K2	−128.3 (2)	K3^iv^—O7B—C7B—K1	−177.60 (16)
Ni1—O1—C7—O7	178.1 (2)	Ni1B—O1B—C7B—O7B	173.5 (3)
K4—O1—C7—O7	28.2 (3)	K1—O1B—C7B—O7B	−30.0 (3)
Ni1—O1—C7—C6	1.8 (3)	Ni1B—O1B—C7B—C6B	−7.7 (3)
K4—O1—C7—C6	−148.1 (2)	K1—O1B—C7B—C6B	148.9 (2)
Ni1—O1—C7—K4	149.87 (15)	Ni1B—O1B—C7B—K1	−156.51 (16)
Ni1—O1—C7—K2	133.7 (7)	O6B—C6B—C7B—O7B	7.6 (5)
K4—O1—C7—K2	−16.1 (8)	N4B—C6B—C7B—O7B	−173.3 (3)
O6—C6—C7—O7	7.3 (4)	K1^iv^—C6B—C7B—O7B	−44.8 (5)
N4—C6—C7—O7	−172.2 (3)	O6B—C6B—C7B—O1B	−171.3 (3)
K2—C6—C7—O7	−8.2 (2)	N4B—C6B—C7B—O1B	7.8 (4)
K3—C6—C7—O7	−55.2 (5)	K1^iv^—C6B—C7B—O1B	136.3 (3)
O6—C6—C7—O1	−176.2 (3)	O6B—C6B—C7B—K1	−72.3 (5)
N4—C6—C7—O1	4.4 (4)	N4B—C6B—C7B—K1	106.7 (4)
K2—C6—C7—O1	168.3 (2)	K1^iv^—C6B—C7B—K1	−124.7 (4)

Symmetry codes: (i) *x*, *y*+1, *z*; (ii) −*x*, −*y*+1, −*z*; (iii) −*x*, −*y*, −*z*; (iv) *x*, *y*−1, *z*; (v) −*x*+1, −*y*+1, −*z*; (vi) −*x*+1, −*y*, −*z*; (vii) *x*, −*y*+1/2, *z*−1/2; (viii) *x*, −*y*+1/2, *z*+1/2.

### Hydrogen-bond geometry (Å, º)

**Table:**

*D*—H···*A*	*D*—H	H···*A*	*D*···*A*	*D*—H···*A*
O8—H8*O*···O9^viii^	0.85	2.02	2.869 (4)	173
O8—H8*P*···O4*B*^ix^	0.85	2.01	2.858 (3)	166
O9—H9*P*···O4^v^	0.86	1.91	2.722 (3)	157
O9—H9*O*···O6*B*^vii^	0.86	2.07	2.864 (3)	153
O10—H10*P*···O4^x^	0.88	2.02	2.887 (3)	168
O10—H10*O*···O7*B*^i^	0.87	2.04	2.882 (3)	164

Symmetry codes: (i) *x*, *y*+1, *z*; (v) −*x*+1, −*y*+1, −*z*; (vii) *x*, −*y*+1/2, *z*−1/2; (viii) *x*, −*y*+1/2, *z*+1/2; (ix) −*x*, *y*+1/2, −*z*+1/2; (x) −*x*+1, *y*+1/2, −*z*+1/2.
